# Addressing present pitfalls in 3D printing for tissue engineering to enhance future potential

**DOI:** 10.1063/1.5127860

**Published:** 2020-02-10

**Authors:** Jesse K. Placone, Bhushan Mahadik, John P. Fisher

**Affiliations:** 1 Biomedical Engineering, West Chester University, West Chester, Pennsylvania 19383, USA; 2 Department of Physics and Engineering, West Chester University, West Chester, Pennsylvania 19383, USA; 3 Fischell Department of Bioengineering, University of Maryland College Park, College Park, Maryland 20742, USA; 4 Center for Engineering Complex Tissues, University of Maryland College Park, College Park, Maryland 20742, USA

## Abstract

Additive manufacturing in tissue engineering has significantly advanced in acceptance and use to address complex problems. However, there are still limitations to the technologies used and potential challenges that need to be addressed by the community. In this manuscript, we describe how the field can be advanced not only through the development of new materials and techniques but also through the standardization of characterization, which in turn may impact the translation potential of the field as it matures. Furthermore, we discuss how education and outreach could be modified to ensure end-users have a better grasp on the benefits and limitations of 3D printing to aid in their career development.

## INTRODUCTION

3D bioprinting has rapidly transformed over the past decade from a niche manufacturing process to a widespread tissue engineering approach. This technology has advanced the development of tissue mimetics with clinical potential, paved the way for developing high-throughput applications for drug discovery, and established tools for understanding different microenvironments in a controlled fashion. However, even with these advances, there are limitations to the application of these studies to tissues and more complex environments. Specifically, in this perspective piece, we will discuss the current state of affairs in 3D printing (3DP) for tissue engineering, its limitations, and ways in which the field can address these issues. We will focus on (1) single and multi-material approaches along with decellularized extracellular matrix (ECM) based bioinks and their broad application to bioprinting; (2) challenges that need to be addressed for its acceptance as a common tool in tissue engineering such as validation (mechanical, chemical, and bioactivity, etc.) as well as repeatability; (3) regulatory concerns and potential solutions; and (4) we will conclude with an insight into how additive manufacturing education could grow to expedite the adoption of this technology in academic, clinical, and commercial settings.

## CURRENT STATE-OF-THE-ART

For tissue engineering applications, there is a drive to develop and fabricate tissues or tissue mimetics for use *in vitro* and *in vivo*. Using an additive manufacturing approach, much emphasis has been placed on systems that are amenable to layer-by-layer construction that can allow for the fabrication of discrete zones such as the zonal architecture of cartilage or for complex geometries.^1–9^ Furthermore, these tissues and mimetics can only become so large without reaching limitations in nutrient and waste diffusion. To address this issue, there has also been an emphasis on manufacturing microvasculature using 3DP^10–12^ and even incorporating features to enhance vascularization *in vivo*.^12^ These tissue mimetics have also been used to model natural processes and barriers such as the placental blood barrier, the blood brain barrier, and the liver.^13–16^ Importantly, model systems can be used to understand key aspects of these niches and provide researchers with a unique point of view that would be unobtainable using traditional *in vivo* or *in vitro* assays. As such, these models will continue to answer fundamental questions regarding the roles of mechanical cues, paracrine signaling, growth factor presentation, and cell–cell interactions in an iterative manner only made possible by the spatial and temporal control provided by additive manufacturing.

However, the driving force behind these major macro-scale tissue and tissue mimetics is the research into various materials used for these applications. Some of these materials, such as polycaprolactone (PCL), poly(lactic-co-glycolic acid) (PLGA), and polystyrene (PS), have been in use for decades, but how they are being processed and modified for use under different printing conditions has allowed for them to evolve with the field.^16–18^ Furthermore, bioinks, either naturally derived or inspired materials for use with biological samples, are rapidly evolving to be used in a variety of applications.^2,16,19–22^ These materials and their development taken in tandem will help drive and facilitate advances in the application of 3D printing to answer questions regarding the key processes in cellular behavior, differentiation, growth, and development.

The choice of bioink and the printing platform used are closely intertwined. A number of additive manufacturing techniques such as extrusion-based, stereolithography, inkjet, and laser-induced forward transfer (LIFT) are commonly used in additive manufacturing (Table I).^4,22–43^ Particularly, there is a high degree of freedom when 3D printing acellular, single-material constructs with either hydrogels or thermoplastics. This is because a wide range of temperature, pressure, and photocrosslinking conditions can be explored using different printing methods. The printability of biocompatible polymers such as poly(lactic-co-glycolic acid) (PLGA),^34^ polycaprolactone (PCL),^36,37^ polystyrene (PS),^44^ and polyurethane^45^ has been extensively characterized. Due to the high melting temperatures and viscosity of thermoplastics, they are typically extrusion-printed at high temperatures and pressures. In these cases, the minimum fiber dimensions can be around a few hundred micrometers as they are limited by the needle size and material properties. Conversely, conventional electrospinning techniques can go down to the tens of micrometers or even nanoscale. However, this process is limited by the presence of harsh solvents required for 3D printing and poor control over architecture.^46^ Recently, the direct-write electrospinning technique has surfaced as an alternative printing platform that allows the user to more precisely control the individual strands similar to extrusion-based printing but at the sub-micrometer scale.^47,48^

**TABLE I.** Comparison of common 3D printing methods. This table outlines key attributes for each of the common printing methods to aid in selecting the best fabrication strategy for a given tissue engineering application.

Method	Example materials	Typical resolution	Key attributes	References
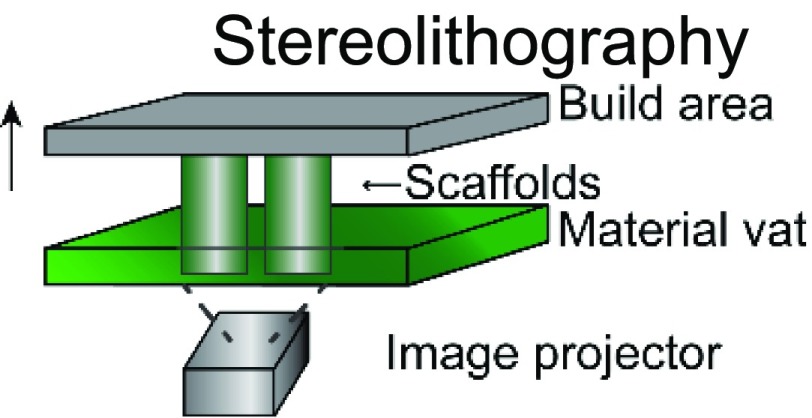	Photocurable resins/inks	∼50 *μ*m	Pros: high speed, well developed technology, low cost, and no viscosity limitations	4, 23–27
			Cons: still limited materials; bioprinting is limited; multi-material printing requires changing out print resins	
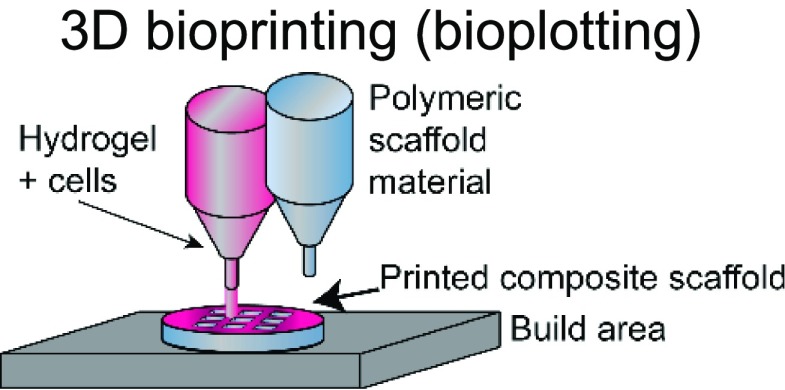	Synthetic and natural polymers, bioinks, and decellularized ECM	∼100–200 *μ*m	Pros: moderate to low cost; high cell density/viability; large number of commercially available printers; and multiple materials can be printed at once	22, 30, 31
			Cons: slow; need for viscous materials; and viability can be affected if shear stress is too high	
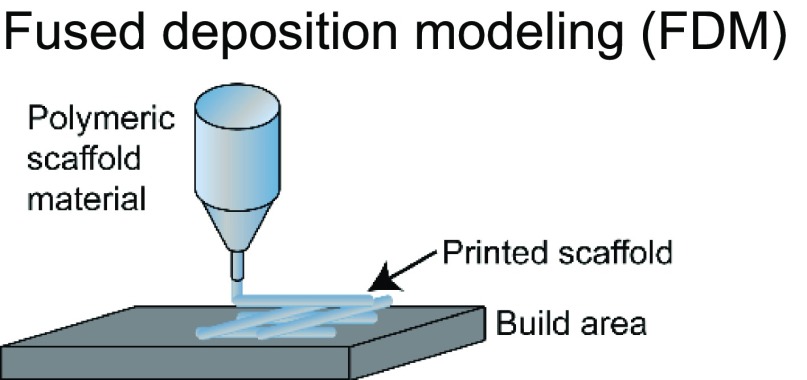	Polymers; thermoplastics	∼100–200 *μ*m	Pros: compatible with a large range of biomaterials and composites; low cost; and can be used concurrently with 3D bioplotting	32–37
			Cons: slow; acellular due to high temperature and pressures; and cells need to be seeded after fabrication	
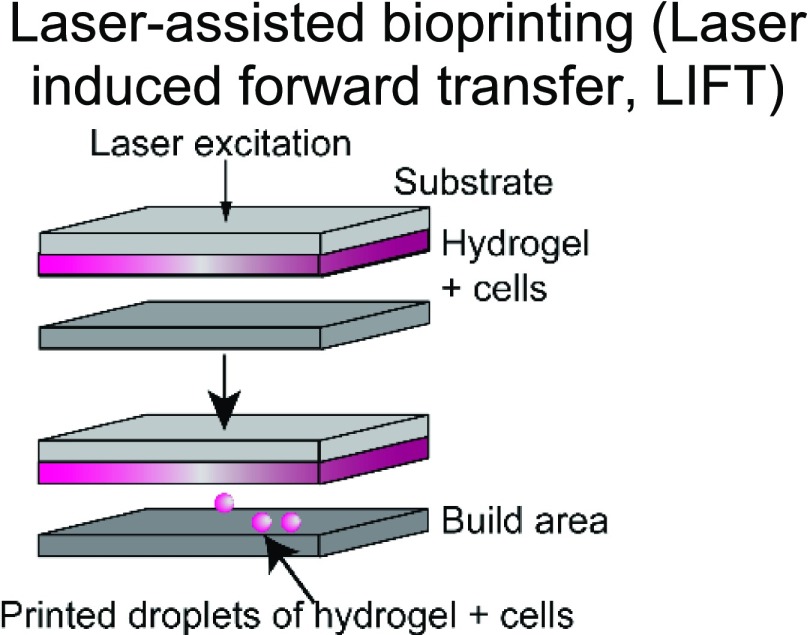	Synthetic and natural polymers	∼10 *μ*m	Pros: high precision/accuracy; can be used with multiple materials and cell types; and can be used to fabricate lab-on-a-chip devices	38–40
			Cons: high cost, long fabrication times; and potentially low cell viability	
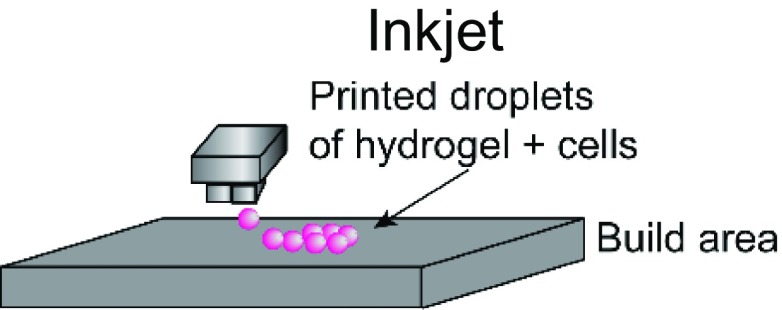	Synthetic and natural polymers	∼10 *μ*m to 100 *μ*m	Pros: low cost; fast fabrication times; and commercially available printers	41–43
			Cons: poor integration between layers and low cell density	

Latest developments in 3D printing also highlight printers (e.g., regenHU) capable of simultaneously producing both extrusion-based and electrospun bioinks, giving researchers access to a broader range of materials, printing conditions, and architecture for 3D printing. Whereas thermoplastics can be cured simply by allowing the strands to cool, hydrogels typically require an additional cross-linking process, either chemical, photochemical, or thermal, to stabilize the resulting structure. Consequently, the desired structure, architecture, and printing strategy are highly dependent on the hydrogel of choice to be 3D printed. Stereolithography-based printing is commonly used for hydrogels that require a light source (visible or UV) to cross-link to create intricate structures using both naturally derived^24,26,27^ and synthetic^25,49^ materials with a high degree of spatial resolution. Polymer viscosity is not a significant barrier in construct fabrication using this technology. However, this platform is more conducive for single-material 3D printing due to its use of a single polymer-containing vat. In contrast, extrusion-based printing enables researchers to print more than one bioink and often a mix of multiple polymers at a single time. The downside is that specific rheological properties are required (such as thixotropy, viscosity, storage, and loss modulus) to create a stable structure after extrusion.^31,50^ Indeed, researchers have explored novel chemistries,^51^ gel-in-gel printing,^52,53^ and polymerization strategies,^54^ to circumvent some of these limitations. Although advantageous, the need for specialized equipment, expensive materials, or highly specific protocols restricts their broad application across 3DP platforms and target tissues.

3DP applications are further complicated by the presence of cells and biologics in the system. To ensure cell viability and activity, the permissible range of temperature, pressure, and cross-linking places constraints on the printing process. For example, incorporation of cells in stereolithography-based vat polymerization is often limited due to the high volumes of polymer solution required and long UV exposure times. Similarly, extrusion printing subjects cells to shear forces that are detrimental to cell viability, making it challenging to print more sensitive cells such as induced pluripotent stem cells (iPSCs) or other stem cells that are easily damaged. Alternatively, inkjet and LIFT bioprinting allow for the printing of polymer solutions with low viscosities and have been used to print a broad range of tissues.^38,43^ These examples illustrate how the desired product may impact the selection of a 3DP fabrication method, or, conversely, how limitation in the fabrication method can restrict tissue design.

Printing platforms have evolved significantly over the past few years as the field of 3D printing has rapidly expanded. For example, to counter the long print times (∼hours) associated with stereolithographic layer-by-layer printing, recent approaches have sought to significantly speed up this process to seconds, potentially enabling scale-up opportunities of biomanufacturing.^55,56^ Similarly, the integration of medical imaging data such as computed tomography (CT) and magnetic resonance imaging (MRI) scans with existing 3D printing platforms has further paved the way for personalized therapies that utilize non-planar extrusion printing to fabricate complex 3D constructs.^57,58^ Other printing techniques such as digital micro-mirror device (DMD) stereolithography,^59^ coaxial 3D printing,^60,61^ and spheroid-based scaffold free fabrication^62^ have further pushed the boundaries of 3D printing applications.

In parallel, a significant amount of research has been devoted to the development of novel multi-material,^63,64^ and hybrid bioinks^65^ that are able to effectively capture the inherent biochemical and biophysical complexity of the native extracellular matrix. The incorporation of multiple materials will allow for these systems to exhibit multiple behaviors.^66^ For instance, to make a bone mimetic, researchers have been utilizing a rigid support material such as thermoplastic polymers in addition to an active, soft material such as a hydrogel bioink to elicit the desired cellular response.^58,67^ However, the inherent mismatch of material properties between these layers or regions leads to poor scaffold performance and failures. Although our knowledge base has progressed with these distinct interfaces, gradients may prove more representative and mimetic of these niches where cells have specific functions in discrete environments. As such, the current trend of looking at interfaces in 3D printing and tightly controlling these interfacial boundaries will prove to be a promising field of research as the printed constructs grow in complexity and function.

The musculoskeletal system is a topic of great interest in interface tissue engineering. It poses a very interesting challenge due to the rapid variations in cellular, mechanical, and ECM composition in a very short spatial range. 3D printing strategies for tissues such as cartilage,^35,68^ the osteochondral interface,^6,69^ and tendons^70–72^ have been well explored to recapitulate their native complexities. As opposed to gradient tissues, skin is a highly complex, stratified tissue that has been well studied in tissue engineering for over two decades. Current clinical treatments for skin regeneration focus mainly on the epidermal layer and are unable to capture the intricate neurovascular, follicular, and sebaceous gland architecture of the dermal and hypodermal layer.^73^ Consequently, 3D printing research has aggressively focused on recreating these distinct, yet interconnected layers in order to provide more meaningful clinical treatments and therapies for patients suffering from severe 2nd and 3rd degree injuries.

Apart from engineering functional tissues, 3D printing also serves several niche applications such as drug delivery,^74,75^ temporal release of drugs and biomolecules,^76^ and biomedical devices. The ability to spatially pattern bioinks allows for the precise incorporation of the desired drugs or biomolecules either on the surface or within the scaffold, while controlling the material degradation rate allows us to control the dosage that ultimately diffuses out of the construct and enters the body. This is in stark contrast to current production techniques for most drugs, which rely on large-scale manufacturing, long production times, and a one-size-fits-all approach. Indeed, with the first Food and Drug Administration (FDA)-approved 3D printed drug Spritam in 2015, the field has opened up toward several drug encapsulation and delivery applications for various tissues.^77–79^ The drug release profile is undoubtedly a critical aspect of drug design and consequently its approval procedure. For example, the active ingredient of Spritam (1000 mg of levetiracetam) disintegrates within seconds after taking a sip of water.^75^ Personalized approaches allow for several potential patient-centric advantages such as tailoring the drug dosage based on age or anatomy, designing its release profile so they have to take pills less frequently, or delivering extremely low or multi-drug doses that combine multiple medications. Key attributes of the established FDA regulatory pathway such as drug safety and efficacy, distribution and metabolism of the active and inactive ingredients, and effects of drug dosage over time can be readily translated to its 3DP counterparts. In order to safely expedite the approval of novel 3DP therapies, several as-yet unanswered aspects of drug design need to be integrated into the established regulatory process for optimal results, such as role of 3DP geometry, material processing, impact of the 3DP technology (powder bed vs extrusion vs stereolithography) on material parameters and drug efficacy, feasibility of the traditional drug screening process on state-of-the-art 3DP drug products, proper evaluation of personalized doses for patients, and presence of any intermediate products during the 3DP process.

## FUTURE GOALS AND TRENDS FOR THE FIELD

Unlike traditional fabrication methods, 3D printing is still in its infancy in terms of the regulations and guidelines for characterizing these materials. As such, researchers typically rely on characterization strategies and ASTM standards developed for other materials/manufacturing techniques. However, more headway needs to be made to establish standards and benchmarks for defining material printability and testing 3D printed constructs and how these constructs are analyzed from a materials perspective. This will help address several of the major problems associated with 3D printing and its adaptation: print fidelity, repeatability, and validation. Once addressed, this manufacturing technique will move from niche applications to play a more ubiquitous role in addressing biological questions and the advancement of engineered tissues.

As the field develops, additional emphasis needs to be placed on creating cell-specific niches where cell-substrate interactions can be tightly controlled. The spatial control afforded by 3D printing will be critical in modulating the cell substrate interactions as a function of position. 3D printing and bioprinting can also be used with materials that behave in a time-dependent manner, thereby allowing for temporal control of the mimetic environment and drug delivery on the requisite time scale for modulating cellular functions.^80–83^ Spatial and temporal control of the local microenvironment will be instrumental in the development of complex, functional tissues where multiple components need to work in concert to drive scaffold remodeling and generate an active tissue. As with the other aspects of 3D printing that are at the forefront of development, establishing clear and uniform methods for assessing the functionality of time dependent materials is critical for their widespread adoption.

With tissue-derived bioinks and decellularized ECM as the base components of 3D printed scaffolds, additional questions will arise. Although these materials are naturally derived, their source and processing conditions may impact the presence and function of cell binding motifs, growth factor binding and presentation, as well as potentially the macroscopic presentation of fibers and their alignment within the printed construct. The impact of these parameters could be substantial on the advancement of these materials for tissue engineering applications.

Commercially available ECM materials such as Matrigel, gelatin, and collagen have lot-to-lot and source dependent variability. The same issues will impact in-house generated materials for 3D printing. However, lot-to-lot variability will impact printability due to material specifications such as the purity, contamination of salts or detergents from the decellularization process, and the degree of substitution, for materials where cross-linking is used to provide structural integrity. Although there are standard protocols for the decellularization process for multiple tissue types, there are many variations and these small changes can impact the reproducibility of published work within and between different research groups. To help alleviate these potential issues, not only should the protocols followed be included in manuscripts, but we also suggest including supplemental information regarding the purity (e.g., salt, detergent, DNA content, etc.) as well as the range of degrees of substitution and molecular weights of the polymers as needed to aid in the adoption of materials by other groups. Journal policies for publication could play an important role in enforcing these requirements.

There are a growing number of commercially available ECM-derived materials for 3D printing, which have been fully characterized from large scientific companies as well as smaller 3D printer start-up companies. Biomaterial kits to be used in 3D printers are becoming more widely available, and the validation for these materials can vary from source-to-source. However, much like the materials developed in-house, it will be critical for the same key parameters to be included with each lot's data sheet due to the impact that even a small perturbation can have on printability. Modulating the material specifications to enhance printability can have undesired consequences that need to be fully understood when translating from *in vitro* to *in vivo* applications. Although *in vitro* systems typically aim to mimic native tissues, translation will remain a significant challenge as the field matures. In particular, considerations need to be taken on how modification for sterility, Good Manufacturing Practices (GMP) compliant materials/production methods, and serum free conditions (recombinant protein and defined culture conditions) can impact the performance of the material as well as cellular response. When implanting in a patient, it is critical to determine and, more importantly prevent any acute inflammatory response to the material, as well as monitor its long-term effects. Consequently, it is often easier to obtain regulatory approval for non-biological implants or for 3DP implants with pre-approved materials. Other aspects of 3DP biological implants such as product integrity after printing, maintaining biomanufacturing consistency with regard to the cellular and biological properties, and sterility at every step of the manufacturing process must be considered. These challenges can potentially create a bottleneck where additional regulatory hurdles need to be overcome for successful transition to the industry/market to address patient needs.

**FIG 1.**
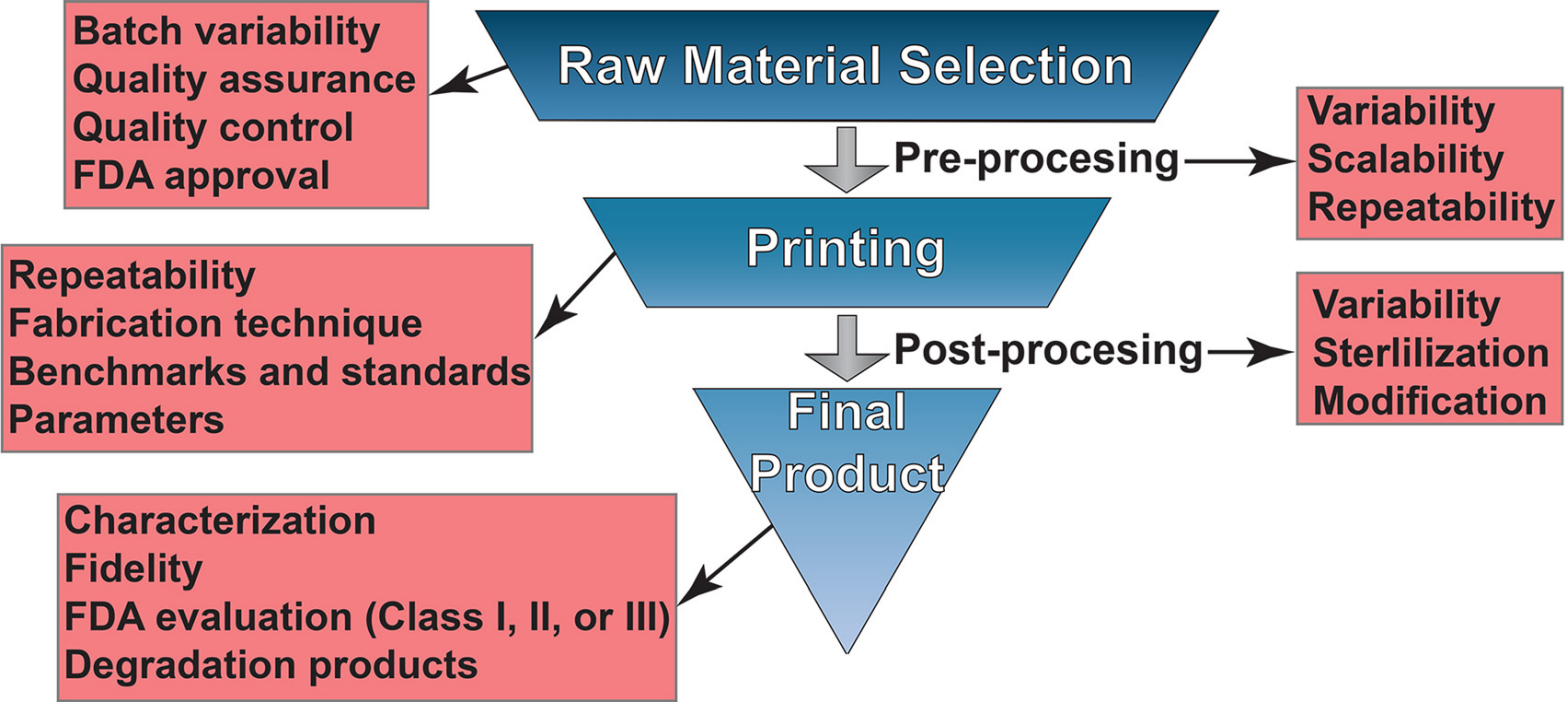
Key steps to generate clinically relevant 3D printed substrates. At each development and fabrication step, researchers need to aid in the development of standards as well as evaluation and characterization methods to ensure repeatability. Consideration needs to be taken with the scale up of each of these steps when transitioning from small scale laboratory settings to larger scale fabrication approaches. Additionally, hands on training and formal education regarding the different parameters that need to be controlled as well as the limitations and constraints on different fabrication strategies will be critical for the continuous adoption of this technology as it matures.

Furthermore, there are different classes of medical devices (e.g., Classes I, II, and III for the FDA) that are regulated differently due to the level of risk they pose to the patient and how much additional validation is required to ensure patient safety and device efficacy.^84^ For 3D printing and additive manufacturing, this adds a level of complexity that needs to be accounted for prior to the fabrication of a new therapeutic device. For instance, if one were to manufacture a Class I device, which has the lowest degree of oversight, there are less stringent guidelines to follow in the device fabrication process. Conversely, a Class III device, such as an implant, would require the most stringent guidelines to make it to market. There, each step of the manufacturing process such as material selection, pre-processing, print parameters (printer type, temperatures, resolution, orientation, etc.), and print post-processing needs to be adequately controlled and verified to provide the best chance of meeting the FDA (or EU Medical Device Regulation) requirements^84,85^ (Fig. 1). As we have discussed throughout this text, we suggest that researchers publish these data in each manuscript's supplementary material section to provide a clear and repeatable method of fabricating a device of interest. This is critical for the long-term adoption of this technology for therapeutic use by maintaining good records at each stage of the process.

A major advantage of 3D printing is the aspect of personalization. The process of translating patient data into a printable product is well established and optimized on the researchers' side. Furthermore, the use of FDA-approved materials for 3D printing and implantation greatly expedites any clinical approvals required for the product to come to the market. However, this represents a conundrum because every patient is unique. Therefore, every stage of the 3D printing process including raw material selection, pre-processing, the actual 3D printing, post-processing, and sterilization prior to implantation have inherent variations that are not necessarily subject to the same regulatory scrutiny (Fig. 1). In other words, the field has yet to address how a scalable biomanufacturing technology that is also capable of individualized treatments can pass important regulatory restrictions in a timely manner. For instance, clinical trials have been carried out on a case-by-case basis due to the regulatory hurdles in several countries using patient-specific medical images to design the implant.^84–86^ The standards and benchmarks associated with 3D printing technologies and 3D printed products, their quality assurance and control (QA/QC), and criteria for success and failure are yet to be fully defined. These are challenges that can only be addressed by the coordinated efforts of the research, clinical, and industrial community. Specifically, as more individual clinical trials are carried out with specific printers and materials, the community needs to work toward developing standards that follow similar production and post-processing procedures. Although the devices are patient-specific, it should be realized that the production methods can be generalized. As these opportunities arise, the community needs to take advantage to facilitate a more timely transition from individual exemptions to widespread clinical adoption and FDA approval.

One critical question also remains: how will these devices be fabricated and/or mass produced? To meet all the necessary requirements, an extensive set of skills will be needed for each step in the production process as described previously and outlined in Fig. 1. A solution to this problem, which if not addressed would hinder and potentially prevent the adoption of 3D printing by the medical community, would be to establish regional centers of excellence for printing implants. Regional 3D printing centers would then develop (1) the expertise needed, (2) the capacity required for the community, (3) the regulatory practices necessary for approval, and (4) the distribution networks to supply the surrounding region (∼100–500 mile radius). Centers of excellence would also be tasked with maintaining adequate records for translation and could act as a repository from which researchers could request information regarding successful material treatments, processing conditions, and designs. Although individual laboratories may be able to undertake a few of the above items, a center devoted to this issue would provide an avenue to accelerate the translational potential of 3D printed medical devices.

Finally, it is important to consider the best utility and application of 3D printing in regenerative medicine. 3D printing is a fantastic technology capable of recapitulating the biology, mechanics, and architecture of native tissue. It has seen its uses in possibly every organ, including bone, skin, muscle, cartilage, heart, liver, kidney, and the neural system. However, it is equally important to consider the complexity associated with printing these structures, their feasibility, and most importantly the value addition compared to standard casting or alternative 3D fabrication methods. It is not uncommon to devote significant time and resources toward 3D printing constructs that represent the desired mimic of the native system. Variations in bioinks, printing protocols, and still-developing regulatory practices further add to the complication. Therefore, it is necessary to ensure that the 3D printed construct represents a truly utilitarian advancement that helps us answer, or pose, questions that would otherwise not be possible via traditional methods. Finally, the barriers to entry owing to the high costs associated with printers, as well as recurring expenses, can be prohibitively high. Indeed, with the recent rise in 3D printing startups and an increasing number of 3D printers entering the market, researchers and clinicians now have a broader range of affordable options.

## EDUCATION

To further aid in the adoption of this technology, education and training are critical. Although there are some maker spaces for inventors and individuals to have access to this technology, the accessibility of the tools needed can be cost prohibitive. However, some groups and institutions are attempting to address this issue through the use of open-source 3D printers that can be built relatively inexpensively. At Carnegie Mellon University (CMU), Dr. Adam Feinberg runs a short course where researchers can come together and build their own open source systems that utilize the Freeform Reversible Embedding of Suspended Hydrogels (FRESH) technology.^87^ Others have taken similar approaches to developing their own printers and have made these available to the community. In addition, there is a cost associated with acquiring patient-specific images for generating 3D models to test our printer capabilities and fabricate patient specific models. Open source repositories such as the NIH 3D print exchange^88^ are one way of addressing this need, but others should be developed to help augment this resource.

In additive manufacturing, the emphasis typically has been on training graduate students either through research experiences or through specific programs, such as the Master of Engineering Program in Additive Manufacturing at the University of Maryland. However, there is still a lack of programs for high school and undergraduate education in additive manufacturing related to tissue engineering problems. To address this issue, some institutions have begun including 3D printers in their machine shops and libraries to encourage students and senior design/capstone teams to make use of this technology. Typically, this does not provide a structured environment for the students to learn more about this very dynamic field. We propose that additional courses and outreach activities need to be encouraged at a university level. For instance, Wake Forest Institute for Regenerative Medicine (WFIRM) and Rice University have outreach programs for high school students to give them hands on experience to encourage them to become engineers in this ever-growing field. The Summer Research Exposure Program (SREP) held by WFIRM is a 6-week training course that pairs students with mentors, giving students direct exposure to tissue engineering research. The Biomaterials Lab at Rice University hosts a week-long internship program for high school students over the summer interested in medicine and research.

Outreach alone will not be enough to address the increase in the applicability of 3D printing to careers in biomedical engineering. As more industry positions need experience with and exposure to 3D printing technology, there will need to be a transition from mainly graduate education (Professional degrees, masters, and Ph.D. level) to undergraduate education. The programs being developed by universities along with commercial entities, such as Cellink and Allevi, to enhance high school and undergraduate student education is critical to fulfilling the need for more technically skilled workers in this growing field.

In practice, the medical community has been one of the first to adopt this technology for training purposes. 3D printed models are seeing use as a way to show medical students pathologies using life size models that they may otherwise not see except as images during their training.^89–91^ Models are not just limited to training but have also been used by surgeons to help plan complex procedures.^92,93^ Training clinicians with 3D printed models and providing life-sized models for surgical planning will improve patient care. As the medical field becomes more familiar with this technology, it is expected that additional strategies including additive manufacturing into patient care will become more commonplace.

Adoption of this technology by industry partners is also key for the long-term growth of this field. We foresee there being a shift at institutional levels to meet the demand for more technically trained students who have familiarity with 3D printing. In academia, traditionally, there has been an emphasis on basic and translational research. However, as more institutions begin developing industry partners, there may be a push for more translational research. There has already been progress in this field in translating specific applications. For instance, high-throughput screening for efficacy and toxicity of therapeutics, drugs, and materials can be made possible by 3D printing strategies.^94^

To continue this trend and increase the success of translating from the bench to commercial applications, the technical knowhow to implement these strategies needs to become available to students across all education levels. This in turn will lead to more career opportunities, and the familiarity with 3D printing technologies will provide realistic expectations for what the technology can bring to the market. Across all fields of tissue engineering currently implementing 3D printing strategies, education will be key for its continuous adoption and acceptance. A firm understanding of the current capabilities of this technology will help drive the adoption of these techniques for clinical and commercial applications.

## CONCLUSIONS

3D bioprinting has seen rapid growth and widespread adoption in recent years. As continuous progress is made in decreasing printer cost and improving printing techniques, material availability, and reproducibility, the adoption of additive manufacturing to more diverse applications in tissue engineering will only continue to climb. Bioinks, natural and artificial ECM based materials, and multi-material-based printing will lead the next generation advances in bioprinting. However, these materials need to be fully characterized beyond just printability to ensure reproducibility and enable their utilization by others. Researchers should place an emphasis on guiding the field toward developing standard techniques and aid in the adoption of standards of regulatory agencies to provide a framework for clinical translation. Establishing centers of 3D printing excellence would facilitate the transition from the bench to clinical applications by localizing the expertise and minimizing the logistical problems that may plague individual groups. In addition, educating the next generation of researchers in the capabilities of this field will place a more realistic expectation and understanding of how these various techniques can be implemented. This will facilitate clear communication on the promise of this technology and avoid over-hyping the technology as a consequence of misunderstanding. As the field continues to mature, addressing these barriers will enable the transition of 3D printing from niche applications to a more widespread technique for 3D culture, high-throughput screening, and device and implant fabrication.

## References

[1] S. V. Murphy and A. Atala , “ 3D bioprinting of tissues and organs,” Nat. Biotechnol. 32(8), 773–785 (2014). 10.1038/nbt.2958 25093879

[2] J. Navarro , G. A. Calderon , J. S. Miller , and J. P. Fisher , “ Bioinks for three-dimensional printing in regenerative medicine,” in *Principles of Regenerative Medicine*, 3rd ed. (Elsevier, 2019), pp. 805–830.

[3] C. Yu , W. Zhu , B. Sun , D. Mei , M. Gou , and S. Chen , “ Modulating physical, chemical, and biological properties in 3D printing for tissue engineering applications,” Appl. Phys. Rev. 5(4), 041107 (2018). 10.1063/1.5050245 31938080 PMC6959479

[4] M. O. Wang , C. E. Vorwald , M. L. Dreher , E. J. Mott , M. H. Cheng , A. Cinar , H. Mehdizadeh , S. Somo , D. Dean , E. M. Brey , and J. P. Fisher , “ Evaluating 3D-printed biomaterials as scaffolds for vascularized bone tissue engineering,” Adv. Mater. 27(1), 138–144 (2015). 10.1002/adma.201403943 25387454 PMC4404492

[5] T. Nie , L. Xue , M. Ge , H. Ma , and J. Zhang , “ Fabrication of poly(L-lactic acid) tissue engineering scaffolds with precisely controlled gradient structure,” Mater. Lett. 176, 25–28 (2016). 10.1016/j.matlet.2016.04.078

[6] S. M. Bittner , B. T. Smith , L. Diaz-Gomez , C. D. Hudgins , A. J. Melchiorri , D. W. Scott , J. P. Fisher , and A. G. Mikos , “ Fabrication and mechanical characterization of 3D printed vertical uniform and gradient scaffolds for bone and osteochondral tissue engineering,” Acta Biomater. 90, 37–48 (2019). 10.1016/j.actbio.2019.03.041 30905862 PMC6744258

[7] J. P. Temple , D. L. Hutton , B. P. Hung , P. Y. Huri , C. A. Cook , R. Kondragunta , X. Jia , and W. L. Grayson , “ Engineering anatomically shaped vascularized bone grafts with hASCs and 3D-printed PCL scaffolds,” J. Biomed. Mater. Res. Part A 102(12), 4317–4325 (2014). 10.1002/jbm.a.35107 24510413

[8] K. Markstedt , A. Mantas , I. Tournier , H. Martínez Ávila , D. Hägg , and P. Gatenholm , “ 3D bioprinting human chondrocytes with nanocellulose–alginate bioink for cartilage tissue engineering applications,” Biomacromolecules 16(5), 1489–1496 (2015). 10.1021/acs.biomac.5b00188 25806996

[9] Y. Lai , Y. Li , H. Cao , J. Long , X. Wang , L. Li , C. Li , Q. Jia , B. Teng , T. Tang , J. Peng , D. Eglin , M. Alini , D. W. Grijpma , G. Richards , and L. Qin , “ Osteogenic magnesium incorporated into PLGA/TCP porous scaffold by 3D printing for repairing challenging bone defect,” Biomaterials 197, 207–219 (2019). 10.1016/j.biomaterials.2019.01.013 30660996

[10] M. D. Sarker , S. Naghieh , N. K. Sharma , and X. Chen , “ 3D biofabrication of vascular networks for tissue regeneration: A report on recent advances,” J. Pharm. Anal. 8(5), 277–296 (2018). 10.1016/j.jpha.2018.08.005 30345141 PMC6190507

[11] D. Lei , Y. Yang , Z. Liu , B. Yang , W. Gong , S. Chen , S. Wang , L. Sun , B. Song , H. Xuan , X. Mo , B. Sun , S. Li , Q. Yang , S. Huang , S. Chen , Y. Ma , W. Liu , C. He , B. Zhu , E. M. Jeffries , F.-L. Qing , X. Ye , Q. Zhao , and Z. You , “ 3D printing of biomimetic vasculature for tissue regeneration,” Mater. Horiz. 6(6), 1197–1206 (2019). 10.1039/C9MH00174C

[12] X. Li , L. Liu , X. Zhang , and T. Xu , “ Research and development of 3D printed vasculature constructs,” Biofabrication 10(3), 032002 (2018). 10.1088/1758-5090/aabd56 29637901

[13] C.-Y. Kuo , A. Eranki , J. K. Placone , K. R. Rhodes , H. Aranda-Espinoza , R. Fernandes , J. P. Fisher , and P. Kim , “ Development of a 3D printed, bioengineered placenta model to evaluate the role of trophoblast migration in preeclampsia,” ACS Biomater. Sci. Eng. 2(10), 1817–1826 (2016). 10.1021/acsbiomaterials.6b00031 33440479

[14] D. Mandt , P. Gruber , M. Markovic , M. Tromayer , M. Rothbauer , S. R. A. Krayz , F. Ali , J. van Hoorick , W. Holnthoner , S. Mühleder , P. Dubruel , S. Van Vlierberghe , P. Ertl , R. Liska , and A. Ovsianikov , “ Fabrication of placental barrier structures within a microfluidic device utilizing two-photon polymerization,” Int. J. Bioprint. 4(2), 144 (2018). 10.18063/IJB.v4i2.144 PMC7581993 33102920

[15] J. A. Kim , H. N. Kim , S.-K. Im , S. Chung , J. Y. Kang , and N. Choi , “ Collagen-based brain microvasculature model in vitro using three-dimensional printed template,” Biomicrofluidics 9(2), 024115 (2015). 10.1063/1.4917508 25945141 PMC4401807

[16] N. Arumugasaamy , L. E. Ettehadieh , C.-Y. Kuo , D. Paquin-Proulx , S. M. Kitchen , M. Santoro , J. K. Placone , P. P. Silveira , R. S. Aguiar , D. F. Nixon , J. P. Fisher , and P. Kim , “ Biomimetic placenta-fetus model demonstrating maternal–fetal transmission and fetal neural toxicity of zika virus,” Ann. Biomed. Eng. 46(12), 1963–1974 (2018). 10.1007/s10439-018-2090-y 30003503

[17] M. Santoro , J. Navarro , and J. P. Fisher , “ Micro- and macrobioprinting: Current trends in tissue modeling and organ fabrication,” Small Methods 2(3), 1700318 (2018). 10.1002/smtd.201700318 30397639 PMC6214196

[18] M. J. Lerman , J. Lembong , G. Gillen , and J. P. Fisher , “ 3D printing in cell culture systems and medical applications,” Appl. Phys. Rev. 5(4), 041109 (2018). 10.1063/1.5046087 PMC7187884 32550961

[19] J. Gopinathan and I. Noh , “ Recent trends in bioinks for 3D printing,” Biomater. Res. 22, 11 (2018). 10.1186/s40824-018-0122-1 29636985 PMC5889544

[20] S. Ji and M. Guvendiren , “ Recent advances in bioink design for 3D bioprinting of tissues and organs,” Front. Bioeng. Biotechnol. 5, 23 (2017). 10.3389/fbioe.2017.00023 28424770 PMC5380738

[21] C. F. Marques , G. S. Diogo , S. Pina , J. M. Oliveira , T. H. Silva , and R. L. Reis , “ Collagen-based bioinks for hard tissue engineering applications: A comprehensive review,” J. Mater. Sci.: Mater. Med. 30(3), 32 (2019). 10.1007/s10856-019-6234-x 30840132

[22] A. Panwar and L. P. Tan , “ Current status of bioinks for micro-extrusion-based 3D bioprinting,” Molecules 21(6), 685 (2016). 10.3390/molecules21060685 PMC6273655 27231892

[23] N. A. Chartrain , C. B. Williams , and A. R. Whittington , “ A review on fabricating tissue scaffolds using vat photopolymerization,” Acta Biomater. 74, 90–111 (2018). 10.1016/j.actbio.2018.05.010 29753139

[24] V. B. Morris , S. Nimbalkar , M. Younesi , P. McClellan , and O. Akkus , “ Mechanical properties, cytocompatibility and manufacturability of chitosan:PEGDA hybrid-gel scaffolds by stereolithography,” Ann. Biomed. Eng. 45(1), 286–296 (2017). 10.1007/s10439-016-1643-1 27164837

[25] Z. Wang , R. Abdulla , B. Parker , R. Samanipour , S. Ghosh , and K. Kim , “ A simple and high-resolution stereolithography-based 3D bioprinting system using visible light crosslinkable bioinks,” Biofabrication 7(4), 045009 (2015). 10.1088/1758-5090/7/4/045009 26696527

[26] Z. Wang , Z. Tian , X. Jin , J. F. Holzman , F. Menard , and K. Kim , “ Visible light-based stereolithography bioprinting cell-adhesive gelatin hydrogels,” in Conference Proceedings–IEEE Engineering in Medicine and Biology Society (2017), p. 159901602. 10.1109/EMBC.2017.8037144 29060188

[27] T. Lam , T. Dehne , J. P. Krüger , S. Hondke , M. Endres , A. Thomas , R. Lauster , M. Sittinger , and L. Kloke , “ Photopolymerizable gelatin and hyaluronic acid for stereolithographic 3D bioprinting of tissue-engineered cartilage,” J. Biomed. Mater. Res., Part B 107, 2649–2657 (2019). 10.1002/jbm.b.34354 PMC6790697 30860678

[28] W. Zhu , X. Ma , M. Gou , D. Mei , K. Zhang , and S. Chen , “ 3D printing of functional biomaterials for tissue engineering,” Curr. Opin. Biotechnol. 40, 103–112 (2016). 10.1016/j.copbio.2016.03.014 27043763

[29] S. Kyle , Z. M. Jessop , A. Al-Sabah , and I. S. Whitaker , “‘ Printability’ of candidate biomaterials for extrusion based 3D printing: State-of-the-art,” Adv. Healthcare Mater. 6(16), 1700264 (2017). 10.1002/adhm.201700264 28558161

[30] D. M. Kirchmajer , R. Gorkin Iii , and M. in het Panhuis , “ An overview of the suitability of hydrogel-forming polymers for extrusion-based 3D-printing,” J. Mater. Chem. B 3(20), 4105–4117 (2015). 10.1039/C5TB00393H 32262288

[31] Y. He , F. Yang , H. Zhao , Q. Gao , B. Xia , and J. Fu , “ Research on the printability of hydrogels in 3D bioprinting,” Sci. Rep. 6, 29977 (2016). 10.1038/srep29977 27436509 PMC4951698

[32] I. Zein , D. W. Hutmacher , K. C. Tan , and S. H. Teoh , “ Fused deposition modeling of novel scaffold architectures for tissue engineering applications,” Biomaterials 23(4), 1169–1185 (2002). 10.1016/S0142-9612(01)00232-0 11791921

[33] M. C. Wurm , T. Möst , B. Bergauer , D. Rietzel , F. W. Neukam , S. C. Cifuentes , and C. Wilmowsky , “ In-vitro evaluation of polylactic acid (PLA) manufactured by fused deposition modeling,” J. Biol. Eng. 11(1), 29 (2017). 10.1186/s13036-017-0073-4 28919925 PMC5594599

[34] T. Guo , T. R. Holzberg , C. G. Lim , F. Gao , A. Gargava , J. E. Trachtenberg , A. G. Mikos , and J. P. Fisher , “ 3D printing PLGA: A quantitative examination of the effects of polymer composition and printing parameters on print resolution,” Biofabrication 9(2), 024101 (2017). 10.1088/1758-5090/aa6370 28244880 PMC5808938

[35] T. Guo , M. Noshin , H. B. Baker , E. Taskoy , S. J. Meredith , Q. Tang , J. P. Ringel , M. J. Lerman , Y. Chen , J. D. Packer , and J. P. Fisher , “ 3D printed biofunctionalized scaffolds for microfracture repair of cartilage defects,” Biomaterials 185, 219–231 (2018). 10.1016/j.biomaterials.2018.09.022 30248646 PMC6186501

[36] A. Guerra , P. Cano , M. Rabionet , T. Puig , and J. Ciurana , “ 3D-printed PCL/PLA composite stents: Towards a new solution to cardiovascular problems,” Materials 11(9), 1679 (2018). 10.3390/ma11091679 PMC6164695 30208592

[37] A. Haryńska , J. Kucinska-Lipka , A. Sulowska , I. Gubanska , M. Kostrzewa , and H. Janik , “ Medical-grade PCL based polyurethane system for FDM 3D printing—Characterization and fabrication,” Materials 12(6), 887 (2019). 10.3390/ma12060887 PMC6471510 30884832

[38] C. S. Ong , P. Yesantharao , C. Y. Huang , G. Mattson , J. Boktor , T. Fukunishi , H. Zhang , and N. Hibino , “ 3D bioprinting using stem cells,” Pediatr. Res. 83(1-2), 223–231 (2018). 10.1038/pr.2017.252 28985202

[39] A. K. Nguyen and R. J. Narayan , “ Liquid-phase laser induced forward transfer for complex organic inks and tissue engineering,” Ann. Biomed. Eng. 45(1), 84–99 (2017). 10.1007/s10439-016-1617-3 27090894

[40] R. Xiong , W. Chai , and Y. Huang , “ Laser printing-enabled direct creation of cellular heterogeneity in lab-on-a-chip devices,” Lab Chip 19(9), 1644–1656 (2019). 10.1039/C9LC00117D 30924821

[41] I. Angelopoulos , M. C. Allenby , M. Lim , and M. Zamorano , “ Engineering inkjet bioprinting processes toward translational therapies,” Biotechnol. Bioeng. 117(1), 272–284 (2019). 31544957 10.1002/bit.27176

[42] E. Masaeli , V. Forster , S. Picaud , F. Karamali , M. H. Nasr-Esfahani , and C. A. Marquette , “ Tissue engineering of retina through high resolution 3-dimentional inkjet bioprinting,” Biofabrication (in press) (2020). 10.1088/1758-5090/ab4a20 31578006

[43] R. Zimmermann , C. Hentschel , F. Schrön , D. Moedder , T. Büttner , P. Atallah , T. Wegener , T. Gehring , S. Howitz , U. Freudenberg , and C. Werner , “ High resolution bioprinting of multi-component hydrogels,” Biofabrication 11(4), 045008 (2019). 10.1088/1758-5090/ab2aa1 31212262

[44] M. J. Lerman , S. Muramoto , N. Arumugasaamy , M. Van Order , J. Lembong , A. G. Gerald , G. Gillen , and J. P. Fisher , “ Development of surface functionalization strategies for 3D-printed polystyrene constructs,” J. Biomed. Mater. Res., Part B 107(8), 2566–2578 (2019). 10.1002/jbm.b.34347 PMC10320806 30821930

[45] S. Y. Jung , S. J. Lee , H. Y. Kim , H. S. Park , Z. Wang , H. J. Kim , J. J. Yoo , S. M. Chung , and H. S. Kim , “ 3D printed polyurethane prosthesis for partial tracheal reconstruction: A pilot animal study,” Biofabrication 8(4), 045015 (2016). 10.1088/1758-5090/8/4/045015 27788126

[46] L. Chen , S. Wang , Q. Yu , P. D. Topham , C. Chen , and L. Wang , “ A comprehensive review of electrospinning block copolymers,” Soft Matter 15(12), 2490–2510 (2019). 10.1039/C8SM02484G 30860535

[47] H. Chen , A. Malheiro , C. van Blitterswijk , C. Mota , P. A. Wieringa , and L. Moroni , “ Direct writing electrospinning of scaffolds with multidimensional fiber architecture for hierarchical tissue engineering,” ACS Appl. Mater. Interfaces 9(44), 38187–38200 (2017). 10.1021/acsami.7b07151 29043781 PMC5682611

[48] T. D. Brown , P. D. Dalton , and D. W. Hutmacher , “ Direct writing by way of melt electrospinning,” Adv. Mater. 23(47), 5651–5657 (2011). 10.1002/adma.201103482 22095922

[49] A. Urrios , C. Parra-Cabrera , N. Bhattacharjee , A. M. Gonzalez-Suarez , L. G. Rigat-Brugarolas , U. Nallapatti , J. Samitier , C. A. DeForest , F. Posas , J. L. Garcia-Cordero , and A. Folch , “ 3D-printing of transparent bio-microfluidic devices in PEG-DA,” Lab Chip 16(12), 2287–2294 (2016). 10.1039/C6LC00153J 27217203 PMC4930360

[50] T. Gao , G. J. Gillispie , J. S. Copus , A. K. Pr , Y.-J. Seol , A. Atala , J. J. Yoo , and S. J. Lee , “ Optimization of gelatin–alginate composite bioink printability using rheological parameters: A systematic approach,” Biofabrication 10(3), 034106 (2018). 10.1088/1758-5090/aacdc7 29923501 PMC6040670

[51] C. Loebel , C. B. Rodell , M. H. Chen , and J. A. Burdick , “ Shear-thinning and self-healing hydrogels as injectable therapeutics and for 3D-printing,” Nat. Protocols 12(8), 1521–1541 (2017). 10.1038/nprot.2017.053 28683063 PMC7546336

[52] A. Basu , A. Saha , C. Goodman , R. T. Shafranek , and A. Nelson , “ Catalytically initiated gel-in-gel printing of composite hydrogels,” ACS Appl. Mater. Interfaces 9(46), 40898–40904 (2017). 10.1021/acsami.7b14177 29091399

[53] T. J. Hinton , Q. Jallerat , R. N. Palchesko , J. H. Park , M. S. Grodzicki , H.-J. Shue , M. H. Ramadan , A. R. Hudson , and A. W. Feinberg , “ Three-dimensional printing of complex biological structures by freeform reversible embedding of suspended hydrogels,” Sci. Adv. 1(9), e1500758 (2015). 10.1126/sciadv.1500758 26601312 PMC4646826

[54] L. Ouyang , C. B. Highley , W. Sun , and J. A. Burdick , “ A generalizable strategy for the 3D bioprinting of hydrogels from nonviscous photo-crosslinkable inks,” Adv. Mater. 29(8), 1604983 (2017). 10.1002/adma.201604983 27982464

[55] P. N. Bernal , P. Delrot , D. Loterie , Y. Li , J. Malda , C. Moser , and R. Levato , “ Volumetric bioprinting of complex living‐tissue constructs within seconds,” Adv. Mater. 31, 1904209 (2019). 10.1002/adma.201904209 31423698

[56] M. P. de Beer , H. L. van der Laan , M. A. Cole , R. J. Whelan , M. A. Burns , and T. F. Scott , “ Rapid, continuous additive manufacturing by volumetric polymerization inhibition patterning,” Sci. Adv. 5(1), eaau8723 (2019). 10.1126/sciadv.aau8723 30746465 PMC6357759

[57] Y.-J. Seol , H. Lee , J. S. Copus , H.-W. Kang , D.-W. Cho , A. Atala , S. J. Lee , and J. J. Yoo , “ 3D bioprinted biomask for facial skin reconstruction,” Bioprinting 10, e00028 (2018). 10.1016/j.bprint.2018.e00028 30911695 PMC6430133

[58] H.-W. Kang , S. J. Lee , I. K. Ko , C. Kengla , J. J. Yoo , and A. Atala , “ A 3D bioprinting system to produce human-scale tissue constructs with structural integrity,” Nat. Biotechnol. 34(3), 312–319 (2016). 10.1038/nbt.3413 26878319

[59] A. K. Miri , D. Nieto , L. Iglesias , H. Goodarzi Hosseinabadi , S. Maharjan , G. U. Ruiz-Esparza , P. Khoshakhlagh , A. Manbachi , M. R. Dokmeci , S. Chen , S. R. Shin , Y. S. Zhang , and A. Khademhosseini , “ Microfluidics-enabled multimaterial maskless stereolithographic bioprinting,” Adv. Mater. 30(27), 1800242 (2018). 10.1002/adma.201800242 PMC6133710 29737048

[60] W. Liu , Z. Zhong , N. Hu , Y. Zhou , L. Maggio , A. K. Miri , A. Fragasso , X. Jin , A. Khademhosseini , and Y. S. Zhang , “ Coaxial extrusion bioprinting of 3D microfibrous constructs with cell-favorable gelatin methacryloyl microenvironments,” Biofabrication 10(2), 024102 (2018). 10.1088/1758-5090/aa9d44 29176035 PMC5837947

[61] X. Liu , S. S. D. Carter , M. J. Renes , J. Kim , D. M. Rojas‐Canales , D. Penko , C. Angus , S. Beirne , C. J. Drogemuller , Z. Yue , P. T. Coates , and G. G. Wallace , “ Development of a coaxial 3D printing platform for biofabrication of implantable islet‐containing constructs,” Adv. Healthcare Mater. 8(7), 1801181 (2019). 10.1002/adhm.201801181 30633852

[62] A. Asakura , Y. Takeoka , K. Matsumoto , D. Taniguchi , T. Tsuchiya , R. Machino , M. Moriyama , S. Oyama , T. Tetsuo , Y. Taura , K. Takagi , T. Yoshida , A. Elgalad , N. Matsuo , M. Kunizaki , S. Tobinaga , T. Nonaka , S. Hidaka , N. Yamasaki , K. Nakayama , and T. Nagayasu , “ Regeneration of esophagus using a scaffold-free biomimetic structure created with bio-three-dimensional printing,” PLos One 14(3), e0211339 (2019). 10.1371/journal.pone.0211339 30849123 PMC6408002

[63] M. Ali , A. K. Pr , J. J. Yoo , F. Zahran , A. Atala , and S. J. Lee , “ A photo‐crosslinkable kidney ECM‐derived bioink accelerates renal tissue formation,” Adv. Healthcare Mater. 8(7), 1800992 (2019). 10.1002/adhm.201800992 PMC7039535 30725520

[64] J. H. Kim , Y.-J. Seol , I. K. Ko , H.-W. Kang , Y. K. Lee , J. J. Yoo , A. Atala , and S. J. Lee , “ 3D bioprinted human skeletal muscle constructs for muscle function restoration,” Sci. Rep. 8(1), 1–15 (2018). 10.1038/s41598-018-29968-5 30120282 PMC6098064

[65] B. J. Klotz , L. A. Oosterhoff , L. Utomo , K. S. Lim , Q. Vallmajo‐Martin , H. Clevers , T. B. F. Woodfield , A. Rosenberg , J. Malda , M. Ehrbar , B. Spee , and D. Gawlitta , “ A versatile biosynthetic hydrogel platform for engineering of tissue analogues,” Adv. Healthcare Mater. 8, 1900979 (2019). 10.1002/adhm.201900979 PMC7116179 31402634

[66] X. Wang , M. Jiang , Z. Zhou , J. Gou , and D. Hui , “ 3D printing of polymer matrix composites: A review and prospective,” Composites, Part B: Eng. 110, 442–458 (2017). 10.1016/j.compositesb.2016.11.034

[67] J. Visser , F. P. W. Melchels , J. E. Jeon , E. M. van Bussel , L. S. Kimpton , H. M. Byrne , W. J. A. Dhert , P. D. Dalton , D. W. Hutmacher , and J. Malda “ Reinforcement of hydrogels using three-dimensionally printed microfibres,” Nat. Commun. 6, 6933 (2015). 10.1038/ncomms7933 25917746

[68] R. Levato , W. R. Webb , I. A. Otto , A. Mensinga , Y. Zhang , M. van Rijen , R. van Weeren , I. M. Khan , and J. Malda , “ The bio in the ink: Cartilage regeneration with bioprintable hydrogels and articular cartilage-derived progenitor cells,” Acta Biomater. 61, 41–53 (2017). 10.1016/j.actbio.2017.08.005 28782725 PMC7116023

[69] A. C. Daly , F. E. Freeman , T. Gonzalez-Fernandez , S. E. Critchley , J. Nulty , and D. J. Kelly , “ 3D bioprinting for cartilage and osteochondral tissue engineering,” Adv. Healthcare Mater. 6(22), 1700298 (2017). 10.1002/adhm.201700298 28804984

[70] T. K. Merceron , M. Burt , Y.-J. Seol , H.-W. Kang , S. J. Lee , J. J. Yoo , and A. Atala , “ A 3D bioprinted complex structure for engineering the muscle–tendon unit,” Biofabrication 7(3), 035003 (2015). 10.1088/1758-5090/7/3/035003 26081669

[71] Y. Wu , Z. Wang , J. Ying Hsi Fuh , Y. San Wong , W. Wang , and E. San Thian , “ Direct E-jet printing of three-dimensional fibrous scaffold for tendon tissue engineering,” J. Biomed. Mater. Res., Part B 105(3), 616–627 (2017). 10.1002/jbm.b.33580 26671608

[72] S. H. Park , Y.-J. Choi , S. W. Moon , B. H. Lee , J.-H. Shim , D.-W. Cho , and J. H. Wang , “ Three-dimensional bio-printed scaffold sleeves with mesenchymal stem cells for enhancement of tendon-to-bone healing in anterior cruciate ligament reconstruction using soft-tissue tendon graft,” Arthroscopy 34(1), 166–179 (2018). 10.1016/j.arthro.2017.04.016 28688825

[73] J. R. Yu , J. Navarro , J. C. Coburn , B. Mahadik , J. Molnar , J. H. Holmes , A. J. Nam , and J. P. Fisher , “ Current and future perspectives on skin tissue engineering: Key features of biomedical research, translational assessment, and clinical application,” Adv. Healthcare Mater. 8(5), 1801471 (2019). 10.1002/adhm.201801471 PMC10290827 30707508

[74] L. K. Prasad and H. Smyth , “ 3D printing technologies for drug delivery: A review,” Drug Dev. Ind. Pharm. 42(7), 1019–1031 (2016). 10.3109/03639045.2015.1120743 26625986

[75] J. Norman , R. D. Madurawe , C. M. V. Moore , M. A. Khan , and A. Khairuzzaman , “ A new chapter in pharmaceutical manufacturing: 3D-printed drug products,” Adv. Drug Delivery Rev. 108, 39–50 (2017). 10.1016/j.addr.2016.03.001 27001902

[76] Y.-Y. Liu , H.-C. Yu , Y. Liu , G. Liang , T. Zhang , and Q.-X. Hu , “ Dual drug spatiotemporal release from functional gradient scaffolds prepared using 3D bioprinting and electrospinning,” Polym. Eng. Sci. 56(2), 170–177 (2016). 10.1002/pen.24239

[77] A. Kjar and Y. Huang , “ Application of micro-scale 3D printing in pharmaceutics,” Pharmaceutics 11(8), 390 (2019). 10.3390/pharmaceutics11080390 PMC6723578 31382565

[78] P. Robles-Martinez , X. Xu , S. J. Trenfield , A. Awad , A. Goyanes , R. Telford , A. W. Basit , and S. Gaisford , “ 3D printing of a multi-layered polypill containing six drugs using a novel stereolithographic method,” Pharmaceutics 11(6), 274 (2019). 10.3390/pharmaceutics11060274 PMC6630370 31212649

[79] X. Farto-Vaamonde , G. Auriemma , R. P. Aquino , A. Concheiro , and C. Alvarez-Lorenzo , “ Post-manufacture loading of filaments and 3D printed PLA scaffolds with prednisolone and dexamethasone for tissue regeneration applications,” Eur. J. Pharm. Biopharm. 141, 100–110 (2019). 10.1016/j.ejpb.2019.05.018 31112767

[80] B. Gao , Q. Yang , X. Zhao , G. Jin , Y. Ma , and F. Xu , “ 4D bioprinting for biomedical applications,” Trends Biotechnol. 34(9), 746–756 (2016). 10.1016/j.tibtech.2016.03.004 27056447

[81] G. H. Yang , M. Yeo , Y. W. Koo , and G. H. Kim , “ 4D bioprinting: technological advances in biofabrication,” Macromol. Biosci. 19(5), e1800441 (2019). 10.1002/mabi.201800441 30821919

[82] N. Ashammakhi , S. Ahadian , F. Zengjie , K. Suthiwanich , F. Lorestani , G. Orive , S. Ostrovidov , and A. Khademhosseini , “ Advances and future perspectives in 4D bioprinting,” Biotechnol. J. 13(12), e1800148 (2018). 10.1002/biot.201800148 30221837 PMC6433173

[83] Y. C. Li , Y. S. Zhang , A. Akpek , S. R. Shin , and A. Khademhosseini , “ 4D bioprinting: The next-generation technology for biofabrication enabled by stimuli-responsive materials,” Biofabrication 9(1), 012001 (2016). 10.1088/1758-5090/9/1/012001 27910820

[84] R. J. Morrison , K. N. Kashlan , C. L. Flanangan , J. K. Wright , G. E. Green , S. J. Hollister , and K. J. Weatherwax , “ Regulatory considerations in the design and manufacturing of implantable 3D-printed medical devices,” Clin. Transl. Sci. 8(5), 594–600 (2015). 10.1111/cts.12315 26243449 PMC4626249

[85] K. Willemsen , R. Nizak , H. J. Noordmans , R. M. Castelein , H. Weinans , and M. C. Kruyt , “ Challenges in the design and regulatory approval of 3D-printed surgical implants: A two-case series,” Lancet Digital Health 1(4), e163–e171 (2019). 10.1016/S2589-7500(19)30067-6 33323186

[86] N. Xu , F. Wei , X. Liu , L. Jiang , H. Cai , Z. Li , M. Yu , F. Wu , and Z. Liu , “ Reconstruction of the upper cervical spine using a personalized 3D-printed vertebral body in an adolescent with Ewing sarcoma,” Spine 41(1), E50–E54 (2016). 10.1097/BRS.0000000000001179 26335676

[87] K. Pusch , T. J. Hinton , and A. W. Feinberg , “ Large volume syringe pump extruder for desktop 3D printers,” HardwareX 3, 49–61 (2018). 10.1016/j.ohx.2018.02.001 30498799 PMC6258044

[88] M. F. Coakley , D. E. Hurt , N. Weber , M. Mtingwa , E. C. Fincher , V. Alekseyev , D. T. Chen , A. Yun , M. Gizaw , J. Swan , T. S. Yoo , and Y. Huyen , “ The NIH 3D print exchange: A public resource for bioscientific and biomedical 3D prints,” 3D Print. Addit. Manuf. 1(3), 137–140 (2014). 10.1089/3dp.2014.1503 28367477 PMC4981148

[89] D. H. Ballard , A. P. Trace , S. Ali , T. Hodgdon , M. E. Zygmont , C. M. DeBenedectis , S. E. Smith , M. L. Richardson , M. J. Patel , S. J. Decker , and L. Lenchik , “ Clinical applications of 3D printing: Primer for radiologists,” Acad. Radiol. 25(1), 52–65 (2018). 10.1016/j.acra.2017.08.004 29030285 PMC6404775

[90] Y. AbouHashem , M. Dayal , S. Savanah , and G. Strkalj , “ The application of 3D printing in anatomy education,” Med. Educ. Online 20, 29847 (2015). 10.3402/meo.v20.29847 26478143 PMC4609651

[91] T. W. Jones and M. D. Seckeler , “ Use of 3D models of vascular rings and slings to improve resident education,” Congenital Heart Dis. 12(5), 578–582 (2017). 10.1111/chd.12486 28608434

[92] N. N. Zein , I. A. Hanouneh , P. D. Bishop , M. Samaan , B. Eghtesad , C. Quintini , C. Miller , L. Yerian , and R. Klatte , “ Three-dimensional print of a liver for preoperative planning in living donor liver transplantation,” Liver Transplant. 19(12), 1304–1310 (2013). 10.1002/lt.23729 23959637

[93] R. J. Mobbs , M. Coughlan , R. Thompson , C. E. Sutterlin , and K. Phan , “ The utility of 3D printing for surgical planning and patient-specific implant design for complex spinal pathologies: Case report,” J. Neurosurg. 26(4), 513 (2017). 10.3171/2016.9.SPINE16371 28106524

[94] M. Vaidya , “ Startups tout commercially 3D-printed tissue for drug screening,” Nat. Med. 21, 2 (2015). 10.1038/nm0115-2 25569539

